# The anorexic hormone Peptide YY_3–36_ is rapidly metabolized to inactive Peptide YY_3–34_ in vivo

**DOI:** 10.14814/phy2.12455

**Published:** 2015-07-21

**Authors:** Signe Toräng, Simon Veedfald, Mette Marie Rosenkilde, Bolette Hartmann, Jens Juul Holst

**Affiliations:** 1 NNF Center for Basic Metabolic Research and Department of Biomedical Sciences, Faculty of Health Sciences, University of CopenhagenCopenhagen, Denmark; 2 Department of Neuroscience and Pharmacology, Faculty of Health Sciences, University of CopenhagenCopenhagen, Denmark

**Keywords:** Degradation, kinetics, Peptide YY

## Abstract

Peptide YY (PYY) is a 36 amino acid peptide hormone released from enteroendocrine cells. An N-terminally degraded metabolite, PYY_3–36,_ has anorexigenic effects, which makes the PYY system a target for obesity treatment. However, little is known about the kinetics and degradation products of PYY. A related peptide, Neuropeptide Y (NPY), may be degraded from the C-terminus. We therefore investigated PYY degradation after in vitro incubations in porcine plasma and blood and in vivo by infusing PYY_3–36_ into multicatheterized pigs (*n* = 7) (2 pmol/kg/min). Plasma samples were analyzed by region-specific radioimmunoassays (RIA) and HPLC analysis. A metabolite, corresponding to PYY_3–34_ was formed after incubation in plasma and blood and during the infusion study. When taking the C-terminal degradation into account, the half-life (*T*½) of PYY in blood and plasma amounted to 3.4 ± 0.2 and 6.2 ± 0.2 h, respectively. After PYY_3–36_ infusion in pigs, the peptide was degraded with a *T*½ of 3.6 ± 0.5 min. Significant extraction (20.5 ± 8.0%) compatible with glomerular filtration was observed across the kidneys and significant C-terminal degradation (26.5 ± 4.8%) was observed across the liver. Net balances across the hind limb, splanchnic bed, and lungs were not significantly different from zero. PYY_3–34_ was unable to activate the Y2 receptor in a transfected cell line. In conclusion, PYY_3–36_ is extensively degraded to PYY_3–34_ in the pig, a degradation that renders the peptide inactive on the Y2 receptor. Currently used assays are unlikely to be able to detect this degradation and therefore measure falsely elevated levels of PYY_3–36_, leading to underestimation of its physiological effects.

## Introduction

Peptide YY (PYY) is a peptide hormone released from a subpopulation of enteroendocrine cells following food intake (Adrian et al. 1985). PYY influences appetite, gastric motility, and water and electrolyte absorption. PYY belongs to a small family of peptides, including PYY, NPY, and Pancreatic Polypeptide (PP). These are all 36 amino acid, C-terminally amidated peptides with high contents of tyrosine, proline, and arginine residues. Their tertiary structure is characterized by a high helical content plus a hairpin structure, often designated “the PP-fold” (Tatemoto 1982; Glover et al. 1984). The family, including PYY binds to and activates Y-receptors. The intact peptide, PYY_1–36_, acts on Y1, Y2, and Y5 receptors, while the N-terminal truncated metabolite, PYY_3–36_, acts on the Y2 receptor. Only the Y2 receptor mediate anorexigenic effects, and therefore only PYY_3–36,_ but not PYY_1–36,_ has attracted interest as a possible drug candidate for obesity treatment (Abbott et al. 1043; Keire et al. 2002).

Peptide YY is synthesized and stored as the 36 amino acid peptide, PYY_1–36_. After its release, the enzyme dipeptidyl peptidase-4 (DPP-4; EC 3.4.14.5), cleaves off two N-terminal residues leading to the formation of the anorexigenic PYY_3–36_ (Eberlein et al. 1989; Medeiros and Turner 1994). PYY_3–36_ is thought to make up 40–55% of total circulating PYY in humans (Grandt et al. 1994), and the formation of PYY_3–36_ can be almost totally blocked by administration of a DPP-4 inhibitor (Aaboe et al. 2010).

**Figure 1:**
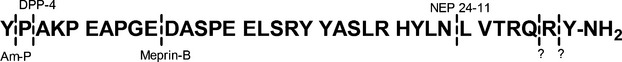
Major cleaving sites of PYY. *Am-P*, aminopeptidase P; *DPP-4*, dipeptidyl peptidase-4; NEP 24-11, neutral endopeptidase 24-11; *?*, proposed cleaving sites.

Besides hydrolysis by DPP-4, in vitro studies showed that PYY can be degraded by aminopeptidase P, neutral endopeptidase 24.11(NEP 24.11), and meprin *β* (Fig.1) (Medeiros and Turner 1994; Addison et al. 2011). However, the importance of these pathways for the metabolism of circulating PYY is unknown. Being a C-terminally amidated peptide, PYY_3–36_ is usually considered relatively protected from C-terminal degradation. However, Abid et al. showed that another peptide from the PP family, NPY, is degraded to NPY_3–35_ in human serum (Abid et al. 2009). In another study, NPY_1–32,_ NPY_1–34_, and NPY_1–35_ were found to be the main metabolites after extensive human plasma incubation (Khan et al. 2007). As PYY and NPY exhibit high homology (70%) and the residues 32–36 are identical, PYY might also be subject to C-terminal degradation.

The measurement of PYY in plasma samples is commonly carried out by RIA or ELISA, using either a specific PYY_3–36_ antibody binding to the truncated N-terminal, or a “side-viewing” antibody which detects both PYY_1–36_ and PYY_3–36_. These assays provide no information about the C-terminus, and the amount of active hormone may therefore be overestimated if a significant C-terminal degradation occurs. NPY being metabolized to NPY_3–35_ and NPY_3–34_ (Khan et al. 2007; Abid et al. 2009) led us to suspect that PYY_3–34_ might be formed in vivo from exogenous as well as endogenous PYY. This study was designed to examine this hypothesis.

## Materials and Methods

This study conformed to the Danish legislation governing animal experimentation, and permission was granted from the National Superintendence for Experimental Animals.

### Anesthetized pigs – exogenous PYY – infusion study

Seven female pigs of the LYD/LYH strain weighing approximately 30 kg were used. Animals were fasted for 16 h but allowed access to drinking water. After premedication with Ketamine (Ketaminol, 10 mg/kg), the animals were anesthetized with *α*-chloralose (Sigma C0128, 100 mg/kg) and ventilated with intermittent positive pressure with N_2_O/O_2_. Vascular catheters were inserted into the pulmonary and a carotid arteries, and into the left femoral, the portal, the hepatic, the left renal, and an ear vein, as described previously (Deacon et al. 1996). After surgery, animals were heparinized and left undisturbed for 30 min.

Synthetic porcine PYY_3–36_ (Bachem H-6042) was dissolved in phosphate buffer with 2% Human serum albumin (HSA, Calbiochem, Merck Millipore, Darmstadt, Germany) and at *t* = 0 a bolus injection (5 pmol/kg) was given in the right ear vein immediately followed up by the infusion of PYY_3–36_ into the right ear vein at a rate of 2 pmol/min/kg using a precision syringe pump. Arterial blood samples were drawn from the carotid artery at −15, −10, 0, 10, 20, 25, 30 min. Between time points 30 and 40 min, two duplicate sets of samples were taken simultaneously from all vascular catheters (carotid artery, pulmonary artery and the femoral, the portal, the hepatic, and the left renal vein). Blood samples were collected into chilled tubes containing EDTA (7.4 mmol/L) and aprotinin (500 KIU/mL), and kept on ice until centrifugation within 20 min. Plasma samples were stored at −20°C until RIA analysis. The PYY infusion was stopped after 45 min and additional arterial blood samples were taken after 2, 5, 10, 15, 20, 30, 45, 60, 85, 115, and 140 min. At *t* = 140 min, 100 pmol/kg PYY_3–36_ was injected and 15 min later blood was sampled for HPLC analysis.

### Anesthetized pigs – endogenous PYY – neuromedin C

Nine female pigs of the LYD/LYH strain weighing approximately 30 kg were anesthetized as described above and catheters placed in the carotid artery and the portal vein. Neuromedin C (120 nmol, Bachem H-3120) was given as a bolus injection at *t* = 0 min, and blood was collected from the carotid artery at −15, −5, 0, 2, 5, 10, 15, 20, 30, 45, and 60 min.

### In vitro degradation of PYY in plasma and blood

Porcine PYY_1–36_ (p- PYY_1–36_) (Bachem H-4505) was incubated in heparinized plasma and whole blood at 37°C for 24 h +/− EDTA (10 mmol/L) and +/− aprotinin (1000 KIE/mL). Samples were taken out regularly and mixed with EDTA, aprotinin and the DPP-4 inhibitor valine pyrrolidide (0.01 mmol/L, final concentration) and frozen instantly, the blood samples after centrifugation, and separation of plasma.

### Hormonal analysis

Plasma samples were analyzed for PYY using two in-house RIAs. Total PYY was measured with a “side-viewing” antibody (T-4093, Bachem), which recognizes PYY_1/3–36_ and PYY_1/3–34_ equally well. As tracer, I^125^ labeled p-PYY_1–36_ was used (NEX240, Perkin Elmer). The C-terminally truncated metabolite was measured using a new antiserum (code no. 2940) raised in white rabbits against residues PYY_23–34_ coupled to keyhole limpet hemocyanin. Synthetic porcine PYY_3–34_ (Genscript, Piscataway, NJ) was used as standard and the tracer was synthetic human PYY_3–34_, ^125^I labeled using stoichiometric chloramine-T method. The assay detects both PYY_1–34_ and_3–34_ and shows negligible cross-reaction with p-PYY_1/3–36_. Assay buffer for both assays was 0.1M tris, pH 8.5, containing 0.2% (wt/vol) HSA, 20 mmol/L EDTA, and 0.6 mmol/L thiomersal (Sigma, St. Louis, MO). Free and bound moieties were separated with plasmacoated charcoal (E. Merck, Darmstadt, Germany). Before PYY measurements plasma was extracted with 70% ethanol (vol/vol, final concentration) to avoid unspecific interference from plasma proteins. Both assays have detection limits below 3 pmol/L (3*standard deviation of the blank) and intraassay variations below 6%.

### High-performance liquid chromatography

Plasma sampled 20 min after the 100 pmol/kg PYY_3–36_ bolus injection was subjected to solid phase extraction (SepPak® Plus tC18, Waters, Milford, MA) and reconstituted in phase A (10% acetonitrile in H_2_O, 0.1% trifluoroacetic acid [TFA]). Plasma was spiked with 0.64 pmol p-PYY_3–36_, p-PYY_3–34_ or p-PYY_1–36_ and loaded onto a HPLC system (Äkta Purifier 900, Amersham Biosciences, Brøndby, Denmark) equipped with a Vydac C_18_ Denali column (238DE54). PYY_3–36_ and PYY_3–34_ were separated by gradient elution (23 to 28% B in 20 min, Phase A: 10% acetonitrile in H_2_O, 0.1% TFA, Phase B: 10% H_2_O in acetonitrile, 0.1% TFA). The flow was set to 1 mL/min and fractions were collected every 12 sec.

### Transfections and tissue culture

COS-7 cells were grown at 10% CO_2_ and 37°C in Dulbeccos modified Eagles medium with GlutaMAX (Invitrogen, Slangerup, Denmark) added 10% fetal bovine serum, 180 U/mL penicillin and 45 *μ*g/mL streptomycin (PenStrep, Thermo Fischer, Slangerup, Denmark). Cotransfection of the hNPY receptor 2 (Y2 receptor) and Gqi4myr was performed using the calcium phosphate precipitation method, as described by Kissow et al. (2012).

### Phosphatidylinositol assay

The cotransfected COS-7 cells were incubated for 24 h with 5 *μ*Ci/mL [myo^3^-H]inositol in growth medium and the assay was carried out as described by Rosenkilde et al. (2005). Three independent experiments with duplicate measurements were performed using increasing concentrations of porcine PYY_3–36_ and porcine PYY_3–34_ (from 10 pmol/L to 1 μmol/L). The dose-response curves were fitted using GraphPad Prism, LaJolla, CA.

### Calculations

Peptide YY_3–36_ concentrations were estimated by subtracting PYY_3–34_ concentrations from total PYY concentrations.

Half-life of PYY in blood and plasma during in vitro incubation was determined from estimated PYY_3–36_ concentrations using the *one phase decay* model of Prism. For each animal, organ extractions of PYY were calculated from arteriovenous concentration differences, as described in detail previously (Deacon et al. 1996).

The in vivo plasma half-life (*T*½) of PYY was determined from the carotid plasma concentrations during the elimination phase (after subtraction of basal arterial concentrations) using the *one phase decay* model of Prism. The metabolic clearance rate was determined from the actual infusion rate divided by the carotid plasma concentration during steady state (mean of plasma concentrations at *t* = 33, 39 and 45 min, basal values subtracted).

### Statistics

Data are expressed as mean ± SEM and analyzed by *t*-test for paired data or one-way ANOVA as appropriate. Differences resulting in *P* < 0.05 were considered significant. Statistical analyses were carried out using GraphPad Prism, version 5.04 for Windows, GraphPad Software).

## Results

### Degradation in vitro

**Figure 2:**
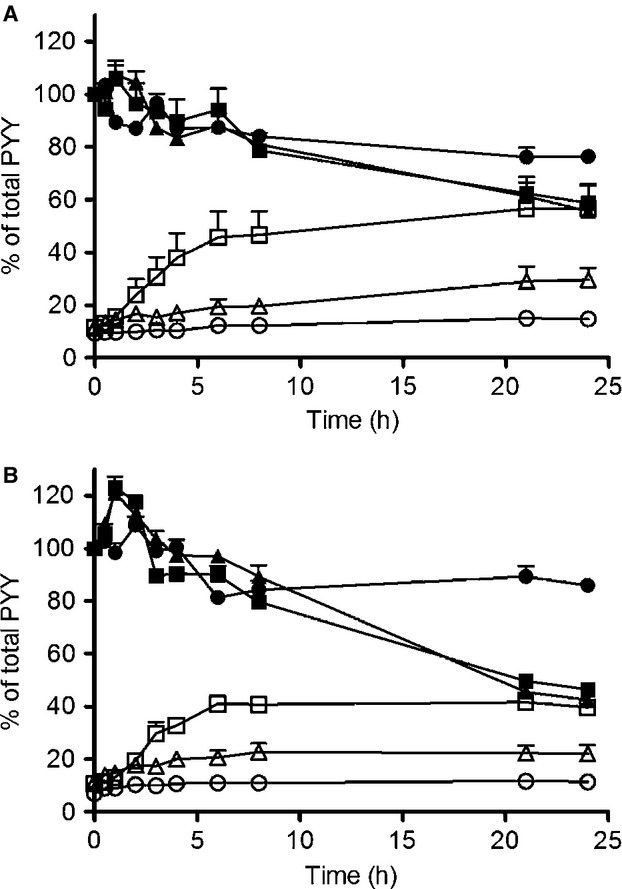
In vitro elimination in plasma. Degradation of PYY_1–36_ in porcine plasma (A) and porcine blood (B). PYY concentrations measured with side-viewing antibody (filled) and PYY_3–34_ C-terminal specific antibody (open). Plasma without added inhibitors (square), plasma + aprotinin (triangle) and plasma + EDTA(circle). Data shown as mean ± SEM, *n*  = 3.

Peptide YY_3–36_ was degraded in blood and plasma in vitro with *T*½ of 3.4 ± 0.2 and 6.2 ± 0.6 h, respectively. In blood, an inexplicable rise in total PYY occurred during the first hour of incubation, and the first two time points were therefore excluded in the determination of half-life. When aprotinin was added to the incubation mixture, the half-life was significantly prolonged to 10.8 ± 0.8 and 11.9 ± 0.7 h, respectively (*P* < 0.05). After addition of EDTA, the degradation no longer followed first-order kinetics, and after 24 h more than 80% of PYY was preserved (Fig.2).

### Metabolism of exogenous PYY_3–36_ in vivo

**Figure 3:**
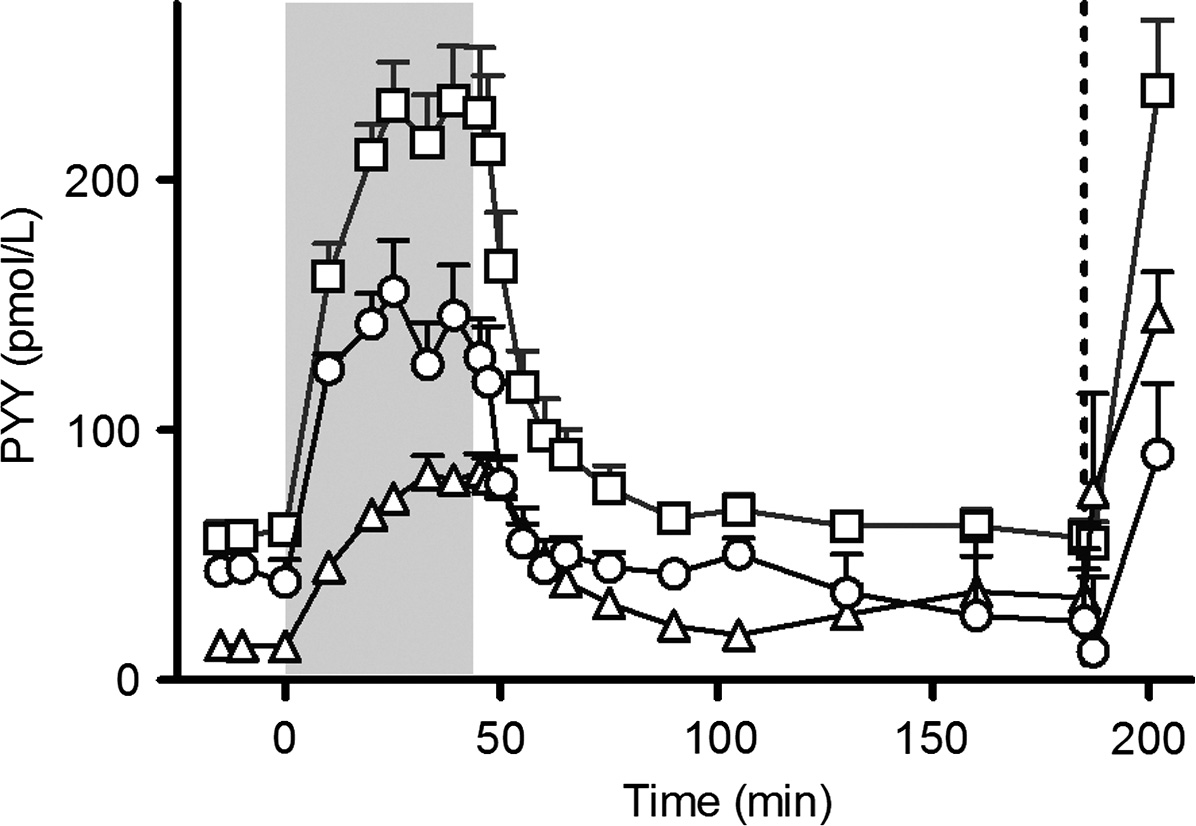
Peptide YY_3–36_ infusion, *t* = 0 to *t* = 45 min (2 pmol/kg/min, gray bar). At *t* = 185 min a bolus injection of 100 pmol/kg PYY_3–36_ was given (dotted line). PYY concentrations measured with side-viewing antibody (square) and PYY_3–34_ C-terminal specific antibody (triangle). Estimated PYY_3–36_ concentrations (circle). Data shown as mean ± SEM,*n*  = 7.

The PYY_3–36_ infusions resulted in a steady state concentration of 228 ± 21 pmol/L total PYY. After the infusion was stopped, PYY was eliminated by first-order kinetics with a *T*½ of 7.3 ± 0.8 min. When corrected for C-terminal degradation (i.e., subtraction of PYY_3–34_ values from total PYY values), the *T*½ was significantly shorter and amounted to 3.6 ± 0.5 min (*P* < 0.05) (Fig.3). Metabolic clearance rate was 12.0 ± 1.2 mL/min/kg calculated from the results of the total PYY RIA but after correction for the C-terminal degradation, the metabolic clearance rate was significantly greater (*P* < 0.01), 28.1 ± 3.7 mL/min/kg.

**Figure 4:**
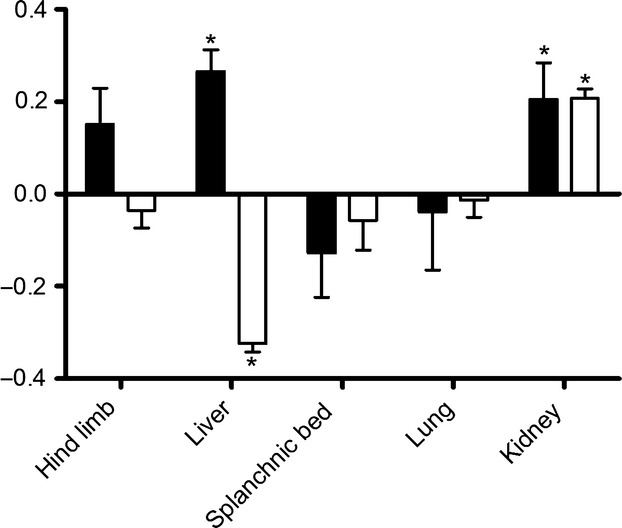
Extraction ratios of PYY_3–36_ (black bars) and PYY_3–34_ (white bars). Negative values indicate net secretion, positive net extraction. *Significantly different from zero, *P* < 0.05. Data shown as mean ± SEM, *n*  = 7.

Significant extraction (20.5 ± 8.0%) of PYY_3–36_ (calculated as PYY_3–34_ subtracted from total PYY), compatible with glomerular filtration was observed across the kidneys, and significant extraction due to C-terminal degradation (26.5 ± 4.8%) was observed across the liver. Net balances across the hind limb, splanchnic bed, and lungs were not significantly different from zero (Fig.4). PYY_3–34_ was also extracted across the kidneys with a ratio compatible with glomerular filtration, but no significant changes were found across the hind limb, splanchnic bed and lungs. Across the liver, a net *formation* (31.4 ± 1.8%) was found (Fig.4).

### Separation of plasma PYY moieties by HPLC

**Figure 5:**
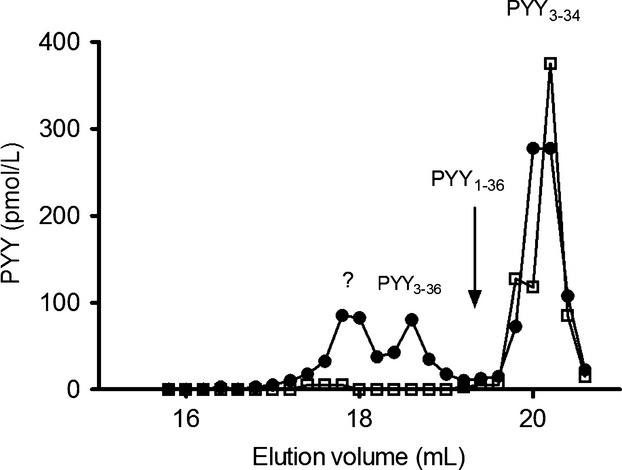
High-performance liquid chromatography separation of PYY_3–36_ and PYY_3–34_ in plasma after injection of 100 pmol/kg PYY_3–36_ in an anesthetized pig. Black circles show immunoreactivity when analyzed with a total PYY assay, open squares PYY_3–34_ immunoreactivity. Different peaks are assigned by analysis of plasma spiked with PYY_1–36_, PYY_3–36_, and PYY_3–34_. The peak marked with *?* is an unknown metabolite recognized by our total PYY assay, but with no PYY_3–34_ immunoreactivity.

After HPLC separation all fractions were subjected to both total PYY and PYY_3–34_ RIA analysis. The total PYY RIA showed three peaks of which two were identified as PYY_3–34_ and PYY_3–36_. The identification was performed by analysis of the same plasma sample, spiked with the three different PYY moieties (p-PYY_1–36_, p-PYY_3–36,_ and p-PYY_3–34_). There was no immunoreactivity at the position of p-PYY_1–36_. The total PYY assay detected an early peak which did not correspond to any of the spike peptides. The PYY_3–34_ assay only recognized the PYY_3–34_ peak (Fig.5).

### Endogenous PYY is degraded to PYY_3–34_

**Figure 6:**
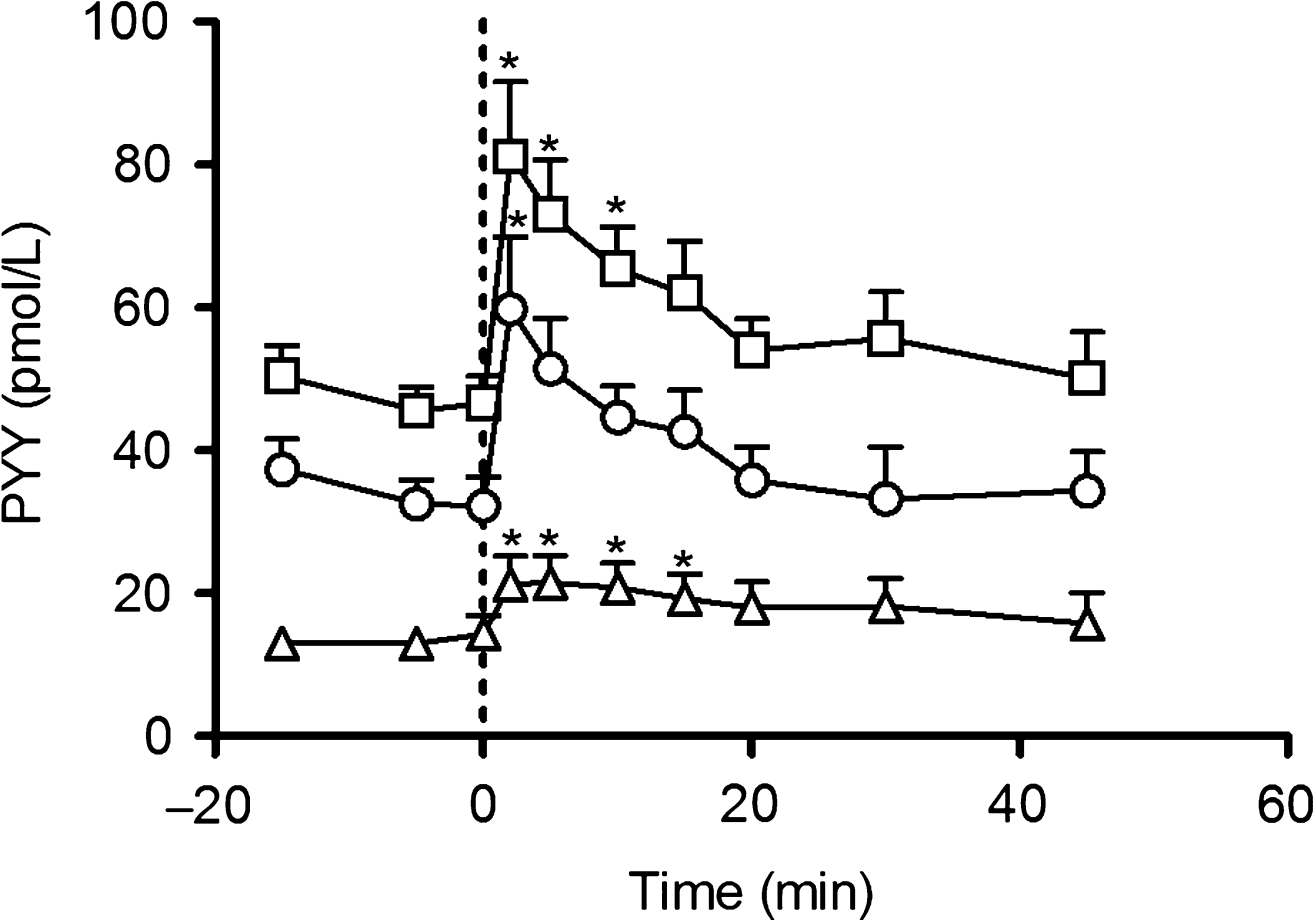
Neuromedin C induced PYY release. At *t* = 0 a bolus injection of 120 nmol neuromedin C was given (dotted line). PYY concentrations measured with side-viewing antibody (square) and PYY_3–34_ C-terminal specific antibody (triangle). Estimated PYY_3–36_ concentrations (circle). *Plasma concentration significantly different from basal level. Data shown as mean ± SEM, *n*  = 9.

After stimulation of PYY secretion with neuromedin C, plasma concentrations of total PYY, PYY_3–36_, and PYY_3–34_ were significantly higher than basal levels. Neuromedin C stimulated PYY release to a peak concentration of total PYY of 81.1 ± 10.5 pmol/L and when corrected for C-terminal degradation to PYY_1/3–34_ the peak concentration was 59.8 ± 10.0 pmol/L (Fig.6).

### PYY_3–34_ does not activate the Y2 receptor

**Figure 7:**
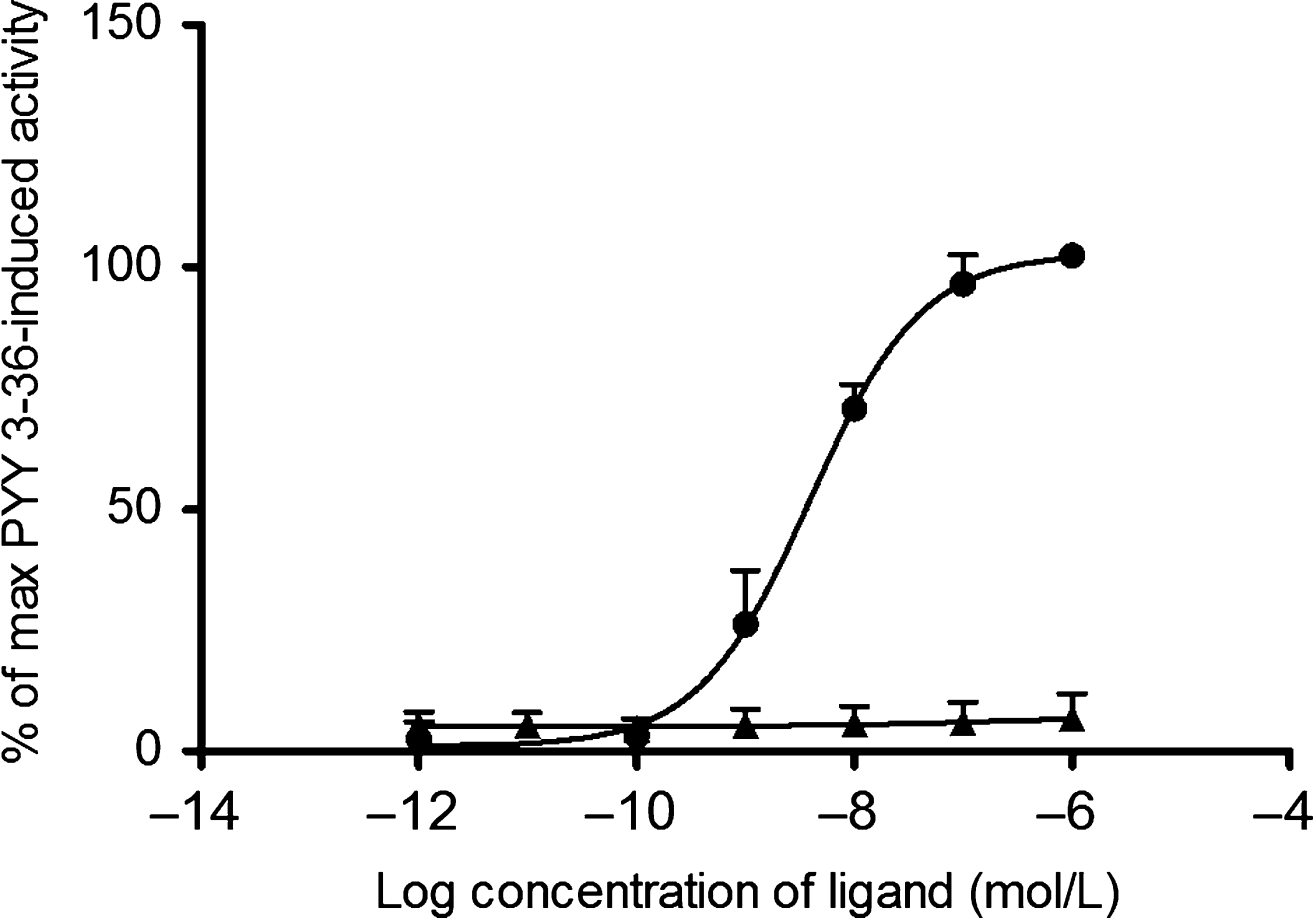
Dose–response curves for IP_3_ generation in COS-7 cells cotransfected with the Y2 receptor and the chimeric G-protein Gqi4myr. The curves are normalized to the maximal activation of the Y2 receptor (upon stimulation with PYY_3–36_). Stimulation with p-PYY_3–36_ (circle) and agonistic (open triangle) and antagonistic (filled triangle) effect of p-PYY_3–34._ Three individual experiments were carried out with duplicate detection. Data shown as mean ± SEM.

In COS-7 cells transiently transfected with the human Y2 receptor and the chimeric G-protein Gqi4myr, we found no phospholipase C activation upon stimulation with PYY_3–34_ (Fig.7). Porcine PYY_3–36_ signaled through the receptor with potency and efficacy similar to human PYY_3–36_ having EC_50_ values of 3.9 nmol/L (Fig.7) and 3.5 nmol/L, respectively.

## Discussion

Recent research has shown that a certain amount of NPY_3–36_ is degraded to NPY_3–35_ when incubated in human plasma (Abid et al. 2009). NPY and PYY are highly homologous and it might therefore be expected that PYY is also degraded from the C-terminus.

In the present investigation, two different assays were used to detect PYY. The antibody used in the assay for total PYY was raised against full-length PYY and detects both PYY_1/3–36_ and PYY_1/3–34_. The antiserum used in the PYY_1/3–34_ assay was raised against PYY_23–34_ and cross-reacts with NPY_1/3–34_, but exhibits less than 1% cross-reaction with PYY_1/3–36._ The cross-reactivity with NPY was not considered a problem, since the concentrations of PYY in the in vitro incubation study were expected to exceed that of (endogenous) NPY many times and in the infusion study, the shape of the PYY_3–34_ curve follows that of the infused PYY_3–36_. When HPLC fractions were analyzed with the two assays, the PYY_3–34_ assay only detected the PYY_3–34_ peak confirming the selectivity of the assay. The total PYY assay detected all the expected peaks (identified by spiking of plasma), but an additional peak was also found. This peak has not been identified, but may reflect another cleavage product of PYY, indicating that the half-life of intact PYY_3–36_ might be shorter than 3.6 ± 0.5 min. We estimated the level of PYY_3–36_ by subtracting PYY_3–34_ from total PYY concentrations. The resulting concentration of such a subtraction is subject to some uncertainty, as the variance of both assays influences the result. Furthermore, it cannot be excluded that the side-viewing antibody binds to other C-terminal degraded moieties that are not recognized by the C-terminal specific assay and vice versa. The only way to overcome this problem is to use a sandwich ELISA with C-terminal and N-terminal specific antibodies, but to our best knowledge assays like this do not exist. Abid et al. found that NPY is degraded to NPY_3–35_, but only detected trace amounts of NPY_3–34_. In contrast, we show here that PYY_3–34_ is a major degradation product of PYY. There may be several reasons for this observed difference. First, although PYY and NPY are very similar, especially with regard to the C-terminal, the two peptides may still be metabolized differently. Second, our in vivo study employed near physiological concentrations, whereas Abid et al. studied degradation in vitro using grossly supraphysiological concentrations which may be handled differently. We also studied the formation of PYY_3–34_ in porcine plasma and blood and found that after 6 h approximately 40% of total immunoactive PYY was C-terminally truncated. This discrepancy could also be due to species differences. It cannot be excluded that the pig metabolizes PYY differently from humans. There certainly is a difference between humans and pigs with regard to both fasting and postprandial PYY, possibly reflecting different eating habits, or another role for PYY in pig physiology (Adrian et al. 1985, 1987).

Abid et al. proposed that the C-terminal tyrosine in NPY is cleaved off by plasma kallikrein. When we incubated PYY in plasma with the kallikrein inhibitor aprotinin, the formation of PYY_3–34_ was inhibited, indicating that kallikrein cleavage of the C-terminal tyrosine residue could be the initial step. Addition of EDTA to the plasma prevented the formation of PYY_3–34_, suggesting that a metalloprotease cleaves off residue 35 or maybe both 35 and 36. In contrast to the cleavage products found by Abid et al., and in agreement with the data presented here on PYY degradation, Khan et al. (2007) incubated NPY (fluorescently labeled at residue 1) in human plasma and found that after 24 h 50% of the peptide was degraded and that the main cleavage products were NPY_1–32_ and NPY_1–34_. When NPY was labeled at residue 4 instead, the main cleavage product was NPY_1–35_, indicating that the N-terminal structure influences C-terminal degradation.

Meprin *β* is reported to hydrolyze PYY between residues 10 and 11 and there are several cleavage sites for NEP 24.11 in PYY. Both enzymes are metalloendoproteases and inhibited by EDTA consistent with the attenuation of total PYY breakdown in the incubation study.

The incubation study also revealed that the breakdown of PYY in both blood and plasma is slow (half-life 3.4 ± 0.2 and 6.2 ± 0.6 h, respectively) and even slower when adding EDTA. Thus, soluble plasma enzymes do not appear to contribute heavily to the degradation of PYY in vivo. Furthermore, the data demonstrate that the risk of PYY degradation after blood sampling in EDTA tubes is small.

The present investigation shows that both total and C-terminally degraded PYY are eliminated by the kidney with an extraction ratio amounting to about 20%, which would be compatible with a loss by glomerular filtration (GFR) assuming that GFR would represent 20% of total renal plasma flow (RPF). GFR has been shown to represent approximately 24–27% of RPF in pigs of same strain and size (Link et al. 1985; Hansen et al. 1998). Thus, glomerular filtration may be the main mechanism for renal elimination of PYY in pigs.

In our study, MCR calculated on the basis of plasma profiles obtained by using the total PYY assay (12.0 ± 1.2 mL/min/kg) amounts to about 4–5 times more than what can be explained by glomerular filtration. There was no significant extraction across the other organs, and degradation by soluble enzymes in blood cannot explain the elimination, as the half-life found after in vitro incubation in blood was 3.4 ± 0.2 h.

Interestingly, our in vivo studies showed that PYY is subjected to considerable C-terminal degradation in the liver, and that this degradation is responsible for a hepatic extraction rate of PYY_3–36_ of 26.5 ± 4.8%. This reveals that the liver and the kidneys are major sites for inactivation/elimination of PYY. An earlier study by Beckh et al., employing in situ perfusion of the rat liver, has shown a lower hepatic extraction of PYY (up to 10% in concentrations up to 500 pmol/L) (Beckh et al. 1992), but the PYY assay they used most likely could not detect C-terminal changes; this would be in accordance with our results with the total PYY assay, where no significant hepatic extraction was detected.

Several studies have shown that the C-terminus is crucial for Y2 receptor activation (Beck-Sickinger et al. 1994; Beck-Sickinger and Jung 1995), and that the loss of the two C-terminal residues most likely interrupt the interaction between PYY and the Y2 receptor.

In accordance with these observations, we found no activity of porcine PYY_3–34_ on the human Y2 receptor.

## Conclusion

In this study, we describe a significant formation of a C-terminally truncated PYY metabolite, PYY_3–34_ as part of the in vivo metabolism of PYY. The currently used PYY assays are unlikely to discriminate between this and intact PYY. Measurements with these assays are therefore likely to overestimate the level of physiologically active hormone, as PYY_3–34_ is not active on the Y2 receptor. Further studies will be required to investigate whether or not the metabolite has other activities.
